# Disease Mapping and Regression with Count Data in the Presence of Overdispersion and Spatial Autocorrelation: A Bayesian Model Averaging Approach

**DOI:** 10.3390/ijerph110100883

**Published:** 2014-01-09

**Authors:** Mohammadreza Mohebbi, Rory Wolfe, Andrew Forbes

**Affiliations:** 1 Biostatistics Unit, Faculty of Health, Deakin University, Melbourne 3125, Australia; 2 Department of Epidemiology and Preventive Medicine, Faculty of Medicine, Nursing and Health Sciences, Monash University, Melbourne 3000, Australia; E-Mails: Rory.Wolfe@monash.edu (R.W.); Andrew.Forbes@monash.edu (A.F.)

**Keywords:** Bayesian variable selection, cancer, disease mapping, ecologic studies, Gibbs sampling, spatial epidemiology

## Abstract

This paper applies the generalised linear model for modelling geographical variation to esophageal cancer incidence data in the Caspian region of Iran. The data have a complex and hierarchical structure that makes them suitable for hierarchical analysis using Bayesian techniques, but with care required to deal with problems arising from counts of events observed in small geographical areas when overdispersion and residual spatial autocorrelation are present. These considerations lead to nine regression models derived from using three probability distributions for count data: Poisson, generalised Poisson and negative binomial, and three different autocorrelation structures. We employ the framework of Bayesian variable selection and a Gibbs sampling based technique to identify significant cancer risk factors. The framework deals with situations where the number of possible models based on different combinations of candidate explanatory variables is large enough such that calculation of posterior probabilities for all models is difficult or infeasible. The evidence from applying the modelling methodology suggests that modelling strategies based on the use of generalised Poisson and negative binomial with spatial autocorrelation work well and provide a robust basis for inference.

## 1. Introduction

For count data, the mean and variance are often related and can be estimated using a single parameter, as in the Poisson model, which is the most frequently used model for analysing disease mapping data. Under this model, the mean and variance of the dependent variable are assumed to be equal, conditional on any variables used to explain differences in the mean across primary sampling units (PSU). In practice, however, this assumption is often false, since the variance can either be larger or smaller than the mean, *i.e.*, both overdispersion and underdispersion can exist in count data.

Statistical methods for analysing spatial patterns of disease incidence or mortality have matured over the past decade or so [1,2,3,4,5]. Selection of the appropriate statistical approach for the analysis of correlated count data is important not only for variance estimation, but also for estimation of the mean [6]. The negative binomial and generalized Poisson (G-Poisson) distributions are frequently used to model count data with overdispersion by inclusion of a second parameter governing the variance specification. These distributions are of interest for modelling count data because they include the Poisson distribution as a special case, and over the range where the second parameter is positive, they are over-dispersed relative to Poisson with a variance to mean ratio exceeding 1. Relationships among these distributions are well known [7,8].

When a count dependent variable’s assumed variance is a function of its mean, one source of overdispersion is due to an inappropriate probability model, for example selecting the Poisson model when the generalised Poisson or negative binomial distribution would better capture the variation [9].

Intra-PSU heterogeneity may induce overdispersion as follows: individuals comprising any population subgroup may differ in terms of characteristics that are known to influence the response and if these characteristics are not included in the set of covariates in a model specification then population heterogeneity across PSUs can lead to extra-Poisson variation in cancer counts [10,11].

Presence of overdispersion is a particular problem for the analysis of geographically correlated data. In addition to the misspecification of the mean function and/ or misspecification of the probability model, spatial autocorrelation is a third cause of overdispersion in geographically correlated count data [4]. For example neighbouring PSUs may tend to have populations that socially, economically and demographically are more alike than non-neighbours or cancer occurrence may have a tendency to cluster.

The purpose of this paper is to consider the problem of modelling cancer counts when overdispersion is likely. We consider spatial regression to estimate the association between relative risk of disease and potential risk factors and map model predicted ratios in which counts in PSUs that are geographically close are assumed to have stronger correlation with each other than counts in PSUs that are geographically dispersed. The development of this work was motivated by our previous study of esophageal cancer (EC) incidence in the Caspian region of Iran during 2001–2005 [12,13].

This paper is structured as follows: in Section 2 we describe the Caspian cancer incidence data set from the Mazandaran and Golestan provinces of Iran and define the data structure and outcome probability models under consideration. This is followed by a description of Bayesian hierarchical models to be employed and an automatic Bayesian covariate selection procedure to evaluate and compare the proposed models. Section 3 presents the results of fitting and comparing the competing models to EC standardised incidence ratios (SIRs) in the Caspian region of Iran using a range of goodness of fit indices. Conclusions and further discussion are presented in Section 4 .

## 2. Methods

### 2.1. Esophageal Cancer Incidence Data in the Caspian Region of Iran

Residents of Mazandaran and Golestan provinces of Iran constituted the study population. The aims of analysis were to determine the extent of spatial variability in risk for esophageal cancer in this area, and to assess the degree to which this variability is associated with socioeconomic status (SES) and dietary pattern indices. During the study period, there were 1,693 EC cases in a population of around 4.5 million people. Population and EC counts were available for the 152 agglomerations in the Mazandaran and Golestan provinces. Geographic coordinates for each agglomeration were also obtained that approximately reflected the geographical centroid of each agglomeration. The distances between agglomeration centres was measured in kilometres and ranged from 9 to 507 km.

**Figure 1:**
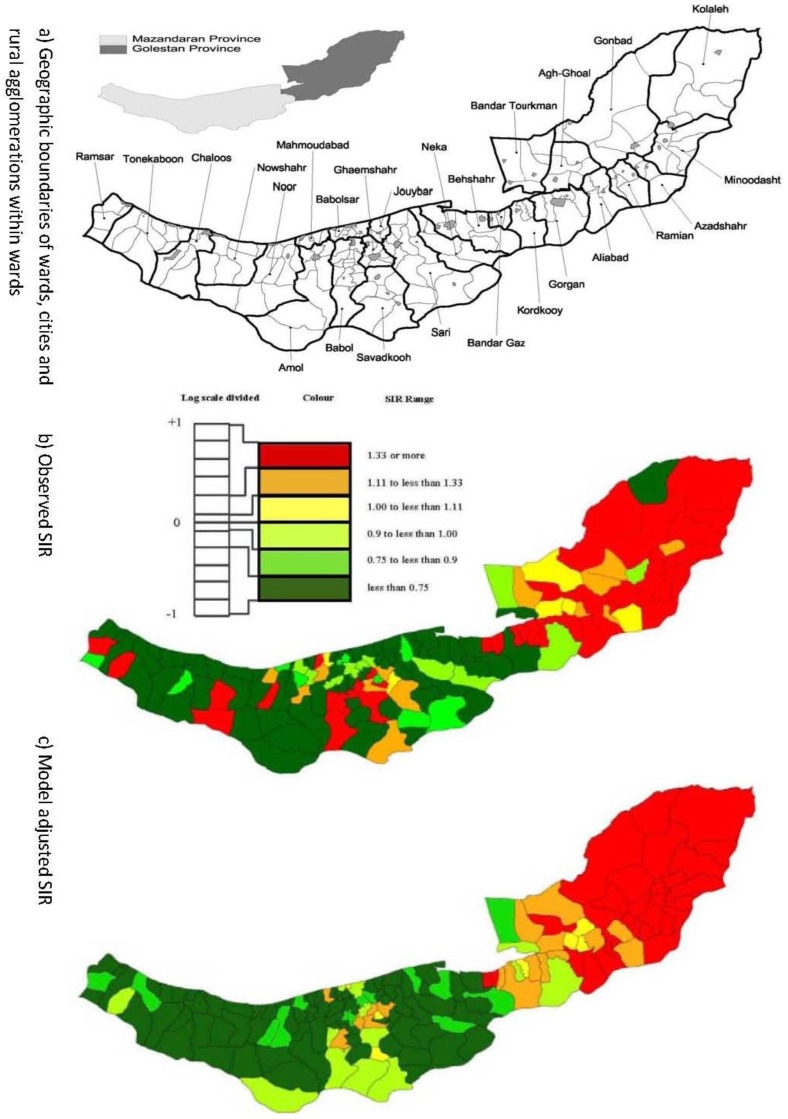
(**a**) Geographic boundaries of wards (bold polygons), cities (grey polygons) and rural agglomerations within wards, in Mazandaran and Golestan provinces; (**b**) Observed spatial pattern; and (**c**) model adjusted SIR.

Figure 1a shows the geographic boundaries of wards, cities and rural agglomerations within wards, in the two provinces. Adjustment of incidence rates for differences in the age structure of agglomerations was accomplished by calculating SIRs with a 2003 population reference. Figure 1b shows strong spatial aggregations among SIR, with a tendency for higher EC rates in the eastern and central agglomerations and lower rates in the west.

Explanatory variables relating to SES were available for each of the 152 agglomerations and to diet for each of the 26 wards [14]. Factor analysis was used to summarise SES and diet variables into a few uncorrelated factors: for SES: “income”, “urbanisation” and “literacy”, with lower values indicating greater deprivation; and for diet: “unrestricted food choice diet” characterized by high intake of foods generally thought to be preventive against EC and “restricted food choice diet” with positive loadings on risky foods. Estimates of the percentage of the population in each ward with diet factor scores in the highest tertile (3rd) were used in regression models. For socio-economic components, factor scores related to each agglomeration were used in the regression model as a continuous covariate. Further details on how the factors were created and defined for diet and SES can be found elsewhere [14].

Log linear models are often used to describe the dependence of the mean function on *k* covariates, *X*_1_, …, *X_k_*. A general form for this type of model for J geographically-defined units (areas) is given by:

*LogE*(*Y_j_*) = *logλ_j_* = *X_j_β^T^* + *θ_j_* + *logE _j_  j = 1*, …, *J* (1)

where *Y_j_* is the count for area j and *E_j_* denotes an “expected” count in area j that is assumed known, *X_j_ =* (1, *X_j_*_1_, …, *X_jk_*) is a 1 × (*k* + 1) vector of area-level risk factors, *β =* (*β*_0_, *β*_1_, …, *β_k_*) is a 1× (*k* + 1) vector of regression parameters and *θ_j_* represents a residual with no spatial structure (so that *θ_i_* and *θ_j_* are independent for *i* ≠ *j*).

### 2.2. Model & Data Structure

The raw data are in the form of disease counts, *Y_j_*, and population counts, *N_j_* in region *j*. The expected count when adjusting for the age structure of an agglomeration, *E_j_*, was obtained by age-standardisation. Then, using the theoretical relationship (*SIR* = 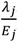).

Equation (1) is equivalent to a model for agglomeration level SIRs. Poisson, generalised Poisson and negative binomial distributions are considered for modelling counts at the agglomeration level and for each of these distributional assumptions, non-spatial, neighbourhood-based and distance-based spatial correlation structures are compared. These analysis approaches are now described in detail.

### 2.3. Distributions for Disease Counts

The Poisson model is given by:

*Y_j_*ǀ(*E_j_*, *λ_j_*) ~ *Poisson*(*λ_j_*) (2)

The Poisson distribution has mean and variance *E*(*Y_j_*) = *V*(*Y_j_*) = *λ_j_*.

The negative binomial, NB, distribution can be constructed by adding a hierarchical element to the Poisson distribution through a random effect *ε_j_*, specifically:

*Y_j_*ǀ(*ε_j_, E_j_, λ_j_*) ~ *Poisson*(*ε_j_λ_j_*), *ε_j_ǀϑ~ gamma*(*ϑ, ϑ*), (3)

for *y_j_* = 0, 1, 2, 3, …, where *ϑ* *>* 0. The resulting probability distribution function marginal to *ε_j_* is:

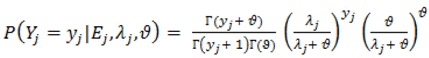

for *y_j_* = 0, 1, 2, 3, …, with *E*(*Y_j_*) = *λ_j_* and *V*(*Y_j_*) = *λ_j_*+ (*λ_j_*)^2^/*ϑ*.

The negative binomial model has the property that the variance is always greater than the mean and *ϑ* is the parameter of extra-Poisson variation with large values of *ϑ* corresponding to variability more like the Poisson distribution. As *ϑ →*∞ the distribution of *Y_j_* converges to a Poisson random variable.

The generalized Poisson, G-Poisson, model with parameters *λ* and *ω* is defined as [9]:

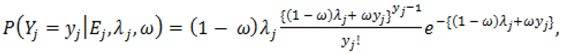
(4)
for *y_j_* = 0, 1, 2, 3, … and has *E*(*Y_j_*) = *λ_j_* and *V*(*Y_j_*) = *λ_j_*(1 − *ω*)^−2^. For *ω* = 0, the generalized Poisson reduces to the Poisson distribution with mean *λ_j_*.

Bayesian inference is based on constructing a model *m* (which encapsulates distributional assumptions and covariate relationships with outcome), its likelihood *f*(*Y*ǀ*γ_m_, m*), and the corresponding prior distribution *f*(*γ_m_*ǀ*m*), where *γ_m_* is a parameter vector under model *m* and *Y* is the outcome variable vector. We use the following hierarchical structure on model parameters:

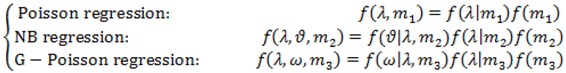
(5)
where *f*(*m*) is the prior probability for entry of covariates in the specification of the linear predictor part of the bigger model *m* within a class of one of the three probability assumptions above.

The maximum total number of candidate models given k covariates (considered additively, *i.e.*, no interactions) is 2^k^. The usual choice for the prior on model *m* is the uniform distribution over the covariate parameter space *M* = {*β*_1_, …, *β_k_*}. We used this uniform distribution because the prior can be thought of as noninformative in the sense of favouring all candidate models equally within the same probability model class.

### 2.4. Hierarchical Models for Relative Risks

Model (1) is a non-spatial model in the sense that it neither recognizes the distance-based relationships among the J agglomerations, nor in area *j* allows for any neighbourhood-based effects between adjacent areas that would mean counts in one area might be related to counts in adjacent areas. Suppose the variability in the {*Y_j_*}*_j_*_ = 1, …,*j*_ follows a spatial model that incorporates assumptions about the spatial relationships between areas. We then extend (1) as:

*LogE*(*Y_j_*) = *logλ_j_* = *X_j_β^T^* + *θ_j_* + *ϕ_j_* + *log E_j_ j* = 1, …, *j* (6)

where the new parameter *ϕ_j_*, represents a residual with spatial structure with *ϕ_i_* and *ϕ_j_*, *i ≠ j*, modelled to have positive spatial dependence. Two approaches are used for modelling the J-dimensional random variable *ϕ*: distance-based and neighbourhood-based spatial correlation structures.

In the distance-based approach the multivariate normal distribution *MVN*(*µ*, *τΣ*) is specified for *ϕ*, where *µ* is a 1 *J* mean vector, *τ* > 0 controls the overall variability of the *ϕ_i_* and *Σ* is a *J* × *J* positive definite matrix. If *d_ij_* denotes the distance between centroids of agglomerations *i* and *j*, then we specify:

∑_ij_ = *f* (*d_ij_; v,k*) (7)

where *f*(*d_ij_; v, k*) = exp[(−*vd_ij_*)^k^]. In this specification *ν* > 0 controls the rate of decrease of correlation with distance, with large values representing rapid decay, and *τ* is a scalar parameter representing the overall precision parameter. The parameter *κ* ϵ (0,2] controls the amount by which spatial variations in the data are smoothed. Large values of *κ* lead to greater smoothing, with *κ* = 2 corresponding to the Gaussian correlation function [15]. The distance-based parameters are jointly referred to as 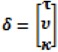.

Besag *et al*. [16] propose modelling the spatial components via a conditional autoregression (CAR) as *ϕ_i_*~N(0, 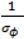) , describing the spatial variation in the heterogeneity component so that geographically close areas tend to present similar risks. One way of expressing this spatial structure is via Markov random fields models where the distribution of each *ϕ_i_* given all the other elements {*ϕ*_1_, …, *ϕ_i_*_ – 1,_ *ϕ_i_*_ + 1_, …, *ϕ_J_*} depends only on its neighbourhood [17]. A commonly used form for the conditional distribution of each *ϕ_i_* is the Gaussian:

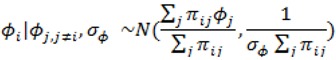
(8)
where the prior mean of each *ϕ_i_* is defined as a weighted average of the other *ϕ_j_*, *j* ≠ *i*, and the weights *π_ij_* define the relationship between area *i* and its neighbours. The precision parameter *σ_ϕ_* controls the amount of variability for the random effect.

Although other possibilities exist, the simplest and most commonly used neighbourhood structure is defined by the existence of a common border of any length between the areas. In this case, the weights *π_ij_* in Equation (8) are constants and specified as *π_ij_* = 1 if *i* and *j* are adjacent and *π_ij_* = 0 otherwise. In that case, the conditional prior mean of *ϕ_i_* is given by the arithmetic average of the spatial effects from its neighbours and the conditional prior variance is proportional to the number of neighbours.

### 2.5. Specification of Priors

In order to be consistent across models with specification of prior belief, the prior distributions imposed on common parameters were the same and non-informative priors were used. A Gamma(0.001, 0.001) prior distribution was used for *ϑ* in the negative binomial distribution, and a Beta(0.5, 0.5) prior for *ω* in the generalized Poisson distribution. The unstructured components were given independent prior distribution 
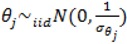
 describing the non-spatial heterogeneity. The hyperparameters *σ_θ_*, *σ_ϕ_* and *δ* are defined below.

### 2.6. Specification of Hyperpriors

In the highest level of the hierarchy prior distributions were specified for the prior precisions for hyperparameters *σ_θ_*, *σ_ϕ_* and *δ*. The estimation of relative risks can be highly dependent on the choice of prior parameters [3] and within a class of Gamma priors, the Gamma(0.5, 0.0005) distribution has been suggested as a sensible choice [2] and was adopted here for the parameters *σ_θ_* and *σ_ϕ_*. For the *δ* parameters a Gamma(0.001, 0.001) prior was used for *τ* and uniform distributions Unif(0.05, 1.95) and Unif(0.05, 20) were used for *κ* and *ν* respectively.

### 2.7. Gibbs Variable Selection, GVS

Candidate models can be represented as (*ψ, α*) ϵ *M* ×{0, 1}^κ^, where *ψ* is a set of binary indicator variables *ψ_g_* (*g* = 1, …, *k*), where *ψ_g_* = 1 or 0 represents respectively the presence or absence of covariate *g* in the model, and *α* denotes other structural properties of the model. For the generalised linear models in this study, *α* describes the distribution, link function, variance function and (un)structured terms, and the linear predictor may be written as:

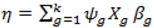
(9)


We assume that α is fixed and we concentrate on the estimation of the posterior distribution of *β* within the class of probability models defined by *α* The prior for (*β*,*ψ*) is specified as *f* (*β*, *ψ*) = *f* (*β*ǀ*ψ*) *f* (*ψ*). Furthermore, *β* can be partitioned into two vectors *β_ψ_* and *β*_\*ψ*_ corresponding to those components of *β* that are included *ψ_g_* = 1 or not included *ψ_g_* = 0 in the model. Then, the prior *f* (*β*ǀ*ψ*) may be partitioned into a “model” prior *f* (*β_ψ_* ǀ*ψ*) and a “pseudo” prior *f* (*β*_\*ψ*_ǀ *β_ψ_, ψ*) [18]. The full posterior distributions for the model parameters are given by:

*f* (*β_ψ_*ǀ*β*_\*ψ*_*, ψ, y*)*~f* (*y*ǀ*β,ψ*) *f* (*β_ψ_*ǀ*ψ*) *f* (*β*_\ψ_ǀ*β*_\*ψ*_*, ψ*) (10)

*f* (*β*_\ψ_ǀ*β*_\*ψ*_*, ψ, y*)~*f* (*β*_\ψ_ǀ*β*_\*ψ*_*, ψ*) (11)

and we assume that the actual model parameters *β_ψ_* and the inactive parameters *β*_\*ψ*_ are a priori independent given *ψ*. This assumption implies that *f* (*β_ψ_*ǀ*β*_\*ψ*_*, ψ, y*)*~f* (*y*ǀ*β,ψ*) *f* (*β_ψ_*ǀ*ψ*) and *f* (*β_\ψ_*ǀ*β*_*ψ*_*, ψ, y*) ∝ *f*(*β_\ψ_*ǀ*ψ*).

The Gibbs sampling procedure is summarized by the following three steps [19]:

(1). Sample the parameters included in the model from the posterior:
  *f* (*β_ψ_*ǀ*β*_\*ψ*_*, ψ, y*) ∝ (*y*ǀ*β,ψ*) *f* (*β_ψ_*ǀ*ψ*)
(2). Sample the parameters excluded from the model from the pseudoprior:
  *f* (*β_\ψ_*ǀ*β*_*ψ*_*, ψ, y*) ∝ *f*(*β_\ψ_*ǀ*ψ*)
(3). Sample each variable indicator j from a Bernoulli distribution with success probability 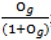; where O*_g_* is given by:

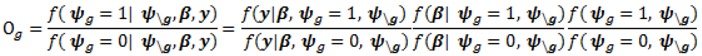
(12)

where *ψ*_\_*_g_* denotes all terms of *ψ* except *ψ_g_*.

The algorithm is further simplified by assuming prior conditional independence of all *β_g_* for each model *ψ*. Then, each prior for *β _g_*ǀ *ψ* consists of a mixture of true prior *f* (*β _g_*ǀ*ψ_g_* = 1*, ψ*_\_*_g_*) for the parameter and a pseudoprior *f* (*β_g_*ǀ *ψ_g_* = 0*, ψ*_\_*_g_*) As a result:

*f* (*β_g_*ǀ *ψ_g_*) = *ψ_g_ f* (*β_g_*ǀ *ψ_g_* = 1) + (1*− ψ_g_*)*f* (*β_g_*ǀ*ψ_g_* = 0) (13)

We considered a normal prior and pseudoprior for the *β_g_s* resulting in:

*f* (*β_g_*ǀ*ψ_g_* = 1)~ *N*(0, *∑_g_*)

and:

*f* (*β_g_*ǀ*ψ_g_* = 0)~ *N*(*µ_G_, S_G_*)

where *µ_G_, S_G_* are the mean and variance respectively in the corresponding pseudoprior distributions and Ʃ*_g_* is the prior variance when covariate g is included in the model.

The Normal prior assumption and Equation (13) result in a prior that is a mixture of two Normal distributions:

*f* (*β_g_*ǀ*ψ_g_*) = *ψ_g_ N*(0*, ∑_g_*) + (1 − *ψ_g_*)*N*(*µ_G_, S_f_*) (14)

Using priors Equation (14) and Equation (9) gives the following full conditional posterior:

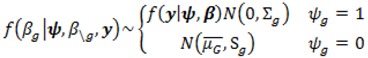
(15)
indicating that the pseudoprior, *f* (*β_g_*ǀ*ψ_g_* = 0) does not affect the posterior distribution of model coefficients.

When no restrictions on the model space are imposed a common prior for the indicator variables *β_g_* is *f*(*ψ_g_*) = Bernoulli (0.5) [20]. The Gibbs sampler was begun with all *ψ_g_* = 1, which corresponds to starting with the full model.

Consider Ʃ as the constructed prior covariance matrix for the whole parameter vector *β* when the multivariate extension of prior distribution (14) is used for each *β_g_*. Zellner’s g prior framework was used to define prior variance structure for Ʃ [21]. The choices *µ_G_* = 0 and S*_g_* = 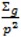 with *p* = 10 were made as they have also been shown to be adequate [18]. The pseudoprior parameters *µ_G_* and *S_g_* are only relevant to the behaviour of the MCMC chain and do not affect the posterior distribution [20].

Because *α* is assumed fixed in our study and we have *k* covariates a set of 2*^K^* competing models are considered *M* = {*m_1_, m_2_, m_3_, …, m_2_k*}*,* and the posterior probability of model *ma* ϵ *M* is defined as:

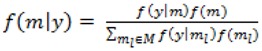
(16)

Bayesian model averaging (BMA) obtains the posterior inclusion probability of a candidate regressor, *pr*(*β_g_*≠ 0ǀ*y*)*, g* = 1*, …, k*, by summing the posterior model probabilities across those regressors that are included in the model.

Within the disease mapping context, usually the aim is prediction. In such cases, prediction should be based on the BMA technique, which also accounts for model uncertainty [22]. Whatever the final intention is (prediction using BMA or selection of a single model) we need to evaluate posterior model probabilities. 

#### 2.7.1. Fully Bayesian Estimation

The Markov chain Monte Carlo method (MCMC) was employed to obtain a sample from the joint posterior distribution of model parameters, automatically generating samples from the marginal posteriors and hyperparameters. It has been suggested that the Gibbs sampler is run for 100,000 iterations for GVS after discarding the first 10,000 iterations for the burn-in period [23]. In our analyses, a total of 500,000 runs with every tenth posterior draw after a burn-in of 50,000 runs was used. The inference of every parameter was thus based on 45,000 posterior samples. Convergence to the posterior distribution was assessed using time series scatterplots, correlograms and the Gelman-Rubin convergence statistic as implemented in WinBUGS and CODA/BOA [24,25].

#### 2.7.2. Comparison of Model Performance

Mean absolute deviance (MAD), mean-squared predictive error (MSPE), pseudo-R^2^ [26], deviance statistic [27], Moran scatter plot [28] and absolute deviance residuals *versus* fitted values [29] were used for estimating the goodness of fit (GOF) and prediction performance of the competing models. Posterior mean of *λ_j_* were used as the plug-in estimate of 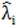 to calculate all the goodness of fit measures discussed in this paper.

Pseudo R^2^ is calculated for model comparison and takes values between zero and one. It is based on 
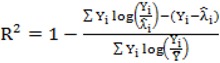
, however since R^2^ increases as more parameters are added to a model regardless of their contribution pseudo R^2^ is defined as Pseudo 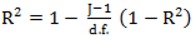 where d.f. for degrees of freedom equal J minus the effective number of free parameters [26].

To assess the prediction performance of the models their mean-squared predictive error and deviance statistic are reported. Mean-squared predictive error is defined as 
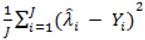
 and mean absolute deviance as 
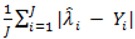
.

The deviance statistic, D= 2{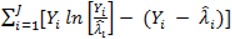} provides evidence of overdispersion as follows: If the deviance index 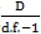 is much greater than 1 this suggests overdispersion. Rules of thumb on the size of the critical threshold vary from 1.2 or 1.3 to as large as 2.0 [30].

The absolute deviance residuals 
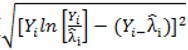
 were plotted against the corresponding fitted values. For a satisfactory specification of the variance function this plot should show a running mean that is approximately straight and flat.

A Moran scatterplot depicts standardised Pearson residuals 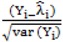 on the horizontal-axis *versus* the spatial lag of the standardised Pearson residual on the vertical axis. The spatial lag averages the effects of the neighbouring spatial agglomerations. By construction, the slope of the line in the scatterplot is equivalent to the Moran’s I coefficient [31]. If the slope is positive it means that there is positive spatial autocorrelation and a negative slope indicates a “checkerboard” spatial pattern.

## 3. Results

The methodology described in Section 2 was applied to the esophageal cancer data from Mazandaran and Golestan.

### 3.1. Automatic Bayesian Model Averaging

The GVS methodology involved covariate selection conditional on the probability distribution and spatial autocorrelation type. With five SES and dietary factors there were 32 covariate models, and hence variable selection was made over the 32 models for a specified probability model type. Posterior summaries of the parameters of interest for the candidate models containing all five covariates are presented in Table 1. The posterior summaries of regression coefficients for models with spatial structure are broadly similar to the nonspatial models. However, 95% credible intervals for regression coefficients in the models that included spatial structure are wider than corresponding intervals in nonspatial models, reflecting the inter-agglomeration correlation being taken into account by the spatial model approaches.

The estimated marginal posterior probabilities were calculated commencing with GVS for all the covariates. Then the covariates were ranked according to the marginal posterior probabilities and factors with marginal posterior inclusion probabilities lower than 0.2 were eliminated, using a rule of thumb [32]. With this approach the following covariates were omitted: unrestricted food choice for non-spatial and neighbourhood-based regressions, and literacy and unrestricted food choice for distance-based regressions. In a second stage, GVS was used again only on the selected covariates from stage one and the subsets created by combinations of these covariates were ranked according to the model posterior probabilities.

BMA of these reduced models was used for prediction purposes. Posterior model probabilities of the top two covariate subsets are presented in Table 2. As Table 2 shows, only income, urbanisation and restricted food choice appeared in the top two covariate subsets. The income and urbanisation factors appeared in all models in at least one of the top two subsets, although the ranking and subsets’ posterior probabilities were slightly different. Urbanisation did not appear in the top two subsets for negative binomial regression with either of the two spatial autocorrelation structures. Table 3 illustrates marginal posterior inclusion probabilities for the top covariate subset of candidate model structure.

**Table 1:** Posterior summaries for Poisson, G-Poisson and Negative Binomial (NB) regression models each with the spatial correlation structures: “IN” independence, “N” neighbourhood-based, “D” distance-based.

Model		Posterior median of regression coefficient β_1_, (95% credible interval)					Random components		
Distribution	Spatial structure	income	urbanisation	literacy	unrestricted food choice	restricted food choice	*σ_θ_*	*σ_ϕ_*	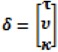
Poisson	IN	−0.22, (−0.60, −0.03)	−0.36, (−0.42, −0.15)	−0.16, (−0.26, −0.08)	0.12, (0.08, 0.16)	−0.32, (−0.41, −0.09)	0.78	-	-
Poisson	IN + N	−0.19, (−0.68, 0.02)	−0.36, (−0.51, −0.16)	−0.15, (−0.22, −0.05)	0.07, (−0.04, 0.16)	−0.24, (−0.38, −0.06)	0.35	0.73	-
Poisson	IN + D	−0.18, (−0.69, 0.07)	−0.35, (−0.51, 0.03)	−0.15, (−0.22, 0.02)	0.07, (−0.03, 0.16)	−0.23, (−0.38, 0.04)	0.13	-	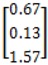
G-Poisson	IN	−0.24, (−0.61, −0.09)	−0.38, (−0.51, −0.09)	−0.18, (−0.22, −0.05)	0.11, (0.09, 0.16)	−0.28, (−0.44, −0.11)	0.56	-	-
G-Poisson	IN + N	−0.19, (−0.69, −0.04)	−0.35, (−0.51, −0.03)	−0.12, (−0.21, −0.03)	0.07, (−0.02, 0.16)	0.23, (−0.38, −0.04)	0.12	0.66	-
G-Poisson	IN + D	−0.19, (−0.68, 0.01)	−0.36, (−0.51, −0.07)	−0.15, (−0.22, 0.06)	0.07, (−0.02, 0.16)	−0.24, (−0.39, −0.07)	0.17	-	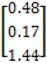
NB	IN	−0.23, (−0.59, −0.10)	−0.39, (−0.58, 0.09)	−0.17, (−0.27, −0.7)	0.17, (0.03, 0.16)	−0.31, (−0.48, −-0.12)	0.36	-	-
NB	IN + N	−0.17, (−0.68−0.06)	−0.35, (−0.51, 0.11)	−0.11, (−0.21, 0.01)	0.07, (−0.04, 0.16)	−0.23, (−0.38, 0.02)	0.12	0.74	-
NB	IN + D	−0.20, (−0.68, 0.10)	−0.35, (−0.51, 0.08)	−0.15, (−0.22, 0.08)	0.07, (−0.01, 0.16)	−0.24, (−0.38, 0.09)	0.11	-	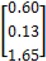

**Table 2:** The top two candidate covariate models (covariate subsets) based on their posterior probabilities: “IN” stands for independence, “N” stands for neighbourhood-based and “D” stands for distance-based structure.

Model distribution	Spatial structure	Subset	Covariates *	f(m|y) **
Poisson	IN	1	income, restricted food choice	0.37
Poisson	IN	2	income, restricted food choice, urbanisation	0.12
Poisson	IN + N	1	income, restricted food choice, urbanisation	0.31
Poisson	IN + N	2	income, restricted food choice	0.15
Poisson	IN + D	1	urbanisation	0.25
Poisson	IN + D	2	income	0.20
G-Poisson	IN + D	2	income, urbanisation	0.18
G-Poisson	IN	1	income, restricted food choice	0.28
G-Poisson	IN	2	income, restricted food choice, urbanisation	0.17
G-Poisson	IN + N	1	income, urbanisation, restricted food choice	0.28
G-Poisson	IN + N	2	urbanisation, restricted food choice	0.13
G-Poisson	IN + D	1	restricted food choice	0.19
G-Poisson	IN + D	2	income, urbanisation	0.18
NB	IN	1	income, restricted food choice	0.21
NB	IN	2	restricted food choice, urbanisation	0.11
NB	IN + N	1	income	0.26
NB	IN + N	2	income, restricted food choice	0.13
NB	IN + D	1	income	0.18
NB	IN + D	2	restricted food choice	0.12

* Covariates are listed in order of decreasing estimated marginal posterior probabilities; ** Posterior probability of the model.

**Table 3:** Marginal posterior inclusion probability for the top candidate models (covariate subsets): “IN” stands for independence, “N” stands for neighbourhood-based and “D” stands for distance-based structure.

Model	Spatial structure	Subset	Covariates	f(*ψ_g_* = 1ǀ*y*) *
distribution				
Poisson	IN	1	income	0.67
			restricted food choice	0.42
Poisson	IN + N	1	income	0.61
			restricted food choice	0.48
			urbanisation	0.37
Poisson	IN + D	1	urbanisation	0.40
G-Poisson	IN	1	income	0.57
			restricted food choice	0.33
G-Poisson	IN + N	1	income	0.59
			urbanisation	0.43
			restricted food choice	0.25
G-Poisson	IN + D	1	restricted food choice	0.22
NB	IN	1	income	0.64
			restricted food choice	0.42
NB	IN + N	1	income	0.47
NB	IN + D	1	income	0.55

* marginal posterior inclusion probability.

### 3.2. Prediction Performance

Table 4 reports the results for the goodness of fit measures used for model comparison based on reduced models that correspond with the covariate subset 1 models in Table 2, retaining only the variables with marginal posterior inclusion probabilities greater than 0.2.

**Figure 2:**
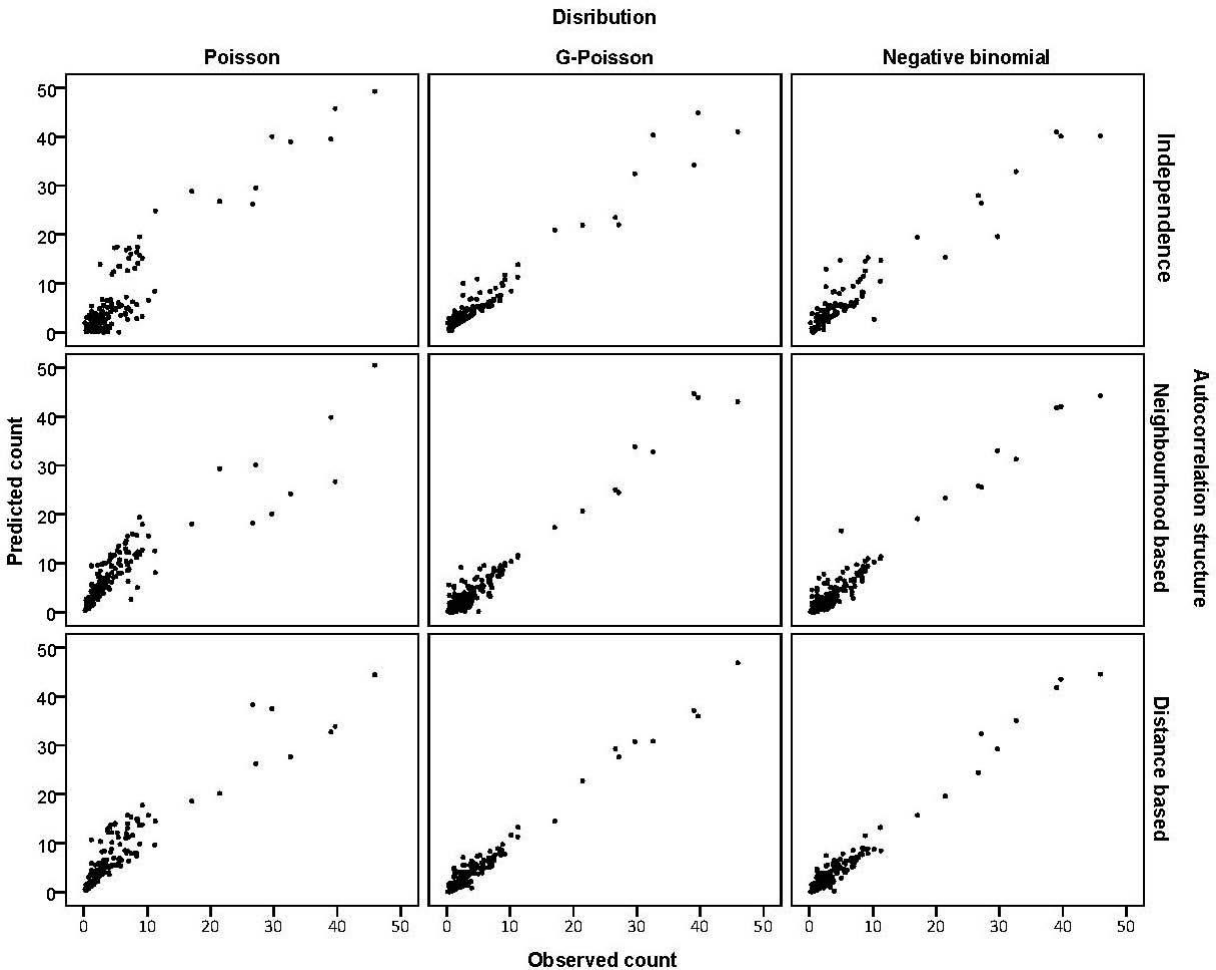
Scatterplots of observed counts (vertical axis) against model predicted counts (horizontal axis) from different models.

The pseudo R^2^ suggested that approximately one third of the total variation in esophageal cancer counts was explained by each of the subset 1 models with slight improvement for joint independence and spatial models. Figure 2 shows the scatterplot of the observed counts against the model predicted counts; consistent with the pseudo R^2^ values the scatterplots show better model fit for spatial models.

**Table 4:** Goodness of fit measures: “IN” stands for independence, “N” stands for neighbourhood-based and “D” stands for distance-based structure.

Model		MAD ^a^	MSPE ^b^	Pseudo-R^2^	Deviance index ^c^
Distribution	Spatial structure				
Poisson	IN	4.4	30.3	0.24	3.1
Poisson	IN + N	3.7	16.6	0.32	2.8
Poisson	IN + D	2.6	13.8	0.28	2.9
G-Poisson	IN	3.2	14.9	0.30	2.6
G-Poisson	IN + N	2.1	10.1	0.35	1.6
G-Poisson	IN + D	2.3	11.6	0.33	1.7
NB	IN	3.4	15.8	0.30	2.4
NB	IN + N	2.2	10.3	0.33	1.7
NB	IN + D	2.3	13.0	0.35	1.4

^a^ Mean absolute deviance; ^b^ Mean-squared predictive error; ^c^ 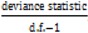

For MSPE and MAD the prediction performances of all spatial models are relatively similar but these spatial models perform better than corresponding non-spatial models. These criteria also suggest that negative binomial and G-Poisson models with neighbourhood-based autocorrelation were preferable to the other models. Figure 1c shows the model adjusted cancer rates from neighbourhood-based negative binomial regression.

### 3.3. Assessing Overdispersion

**Figure 3:**
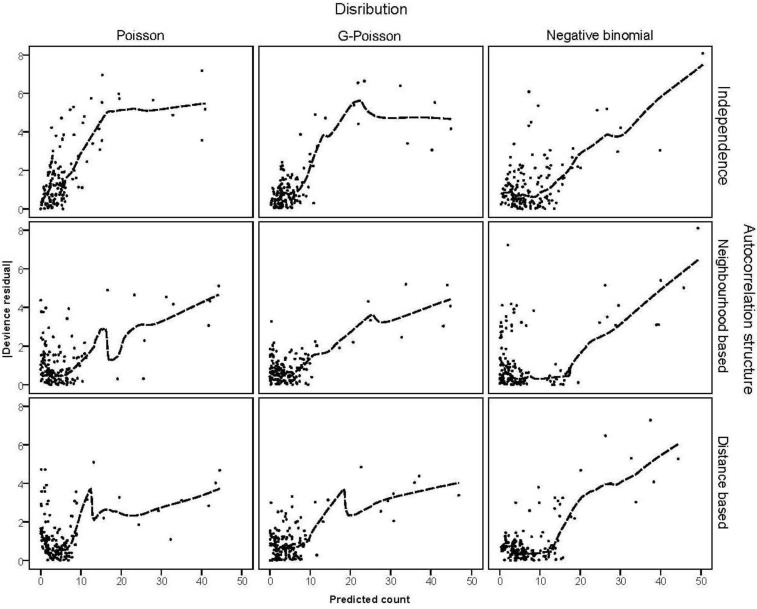
Absolute deviance residuals *versus* fitted values from competing models.

The deviance statistic is reported in Table 4 to provide evidence of overdispersion. Poisson models clearly show overdispersion, as do independence structures in the generalised Poisson and negative binomial models. The deviance measure divided by the d.f.-1 is less than 2 for the generalised Poisson and negative binomial models with spatial correlation structures. Figure 3 presents the absolute deviance residuals plotted against the corresponding fitted values. Figure 3 shows an upward trend, indicating that the assumed variance function is not increasing sufficiently fast with the mean. The running mean for trend is overly sensitive to the points at the extremes, so we suggest concentrating on the central part of the graphs. The plots demonstrate that all models do reasonably well while it is hard to distinguish between the competing models on the basis of this index.

### 3.4. The Moran Scatterplots

**Figure 4:**
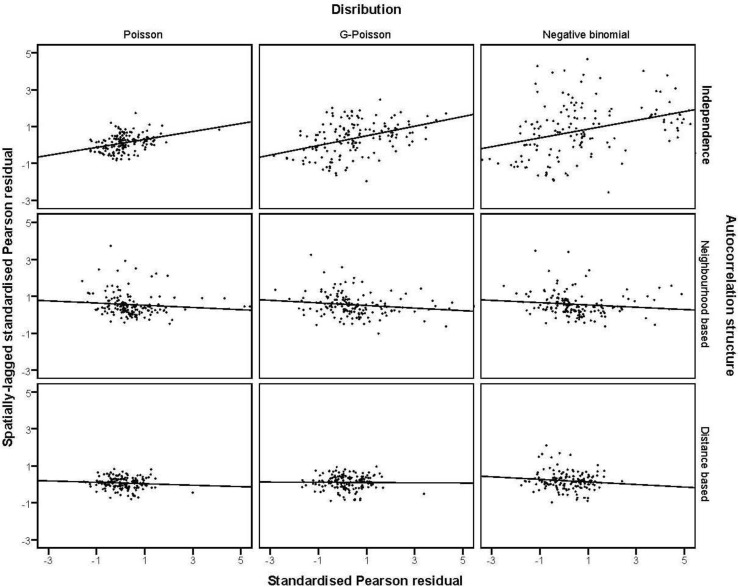
Moran scatter plot of the residuals from competing models: standardised Pearson residuals against spatially-lagged standardised Pearson residuals.

Moran scatterplots in Figure 4 suggest that there is positive spatial autocorrelation in the Pearson residuals in non-spatial models. However the scatterplots for regressions with neighbourhood-based and distance-based structures in Figure 4 suggest that residual spatial autocorrelation is no longer a problem.

## 4. Discussion

Bayesian techniques are recognised as powerful tools in disease mapping but little is known about how these methods compare when applied to real data. Reviews and comparison of Bayesian hierarchical and/or non-hierarchical methods suggested for the analysis of aggregate count data in the context of disease mapping and spatial regression can be found in [2,4,33,34,35].

Our study aims were to assess the risk factors of EC cancer using an automatic Bayesian covariate selection procedure, and to compare prediction performance of the competing models using three distributions for modelling count data to deal with overdispersion and three spatial correlation structures to take account of intra- and inter-agglomeration variation. In conclusion, the use of joint models that include both spatial and nonspatial random effects gave a better picture in terms of model goodness of fit and prediction performance. Generalised Poisson and NB models also performed better than Poisson regression. Overall, generalised Poisson or NB models with conditional autoregressive (CAR) correlation structure seemed to provide the most satisfactory basis for inference.

Two spatial structures were considered in our models: the neighbourhood-based autocorrelation structure that borrows strength from neighbouring agglomerations and the distance-based autocorrelation structure that borrows strength from agglomerations over an effective range. The use of the spatial term resulted in more conservative estimates by explicitly modelling the positive inter-agglomeration correlation of the SIRs, compared with the models that ignored this inter-agglomeration correlation. A nonspatial random effect was included along with spatial random effects to take into account agglomeration heterogeneity. The nonspatial term is especially important in CAR structure, because if the majority of the variability is nonspatial, inference for the CAR model might incorrectly suggest that spatial dependence was present. Results from a simulation study have indicated that if the data are truly independent, a model with CAR random effects and no nonspatial random effects leads to very poor efficiency in the estimation of regression coefficients [36].

In model selection the uniform prior distribution on model space is typically used by setting 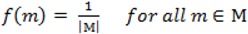. When using the variable selection indicators *ψ*, this prior is equivalent to specifying independent Bernoulli prior distribution with inclusion probability equal to 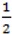. Although *ψ* = 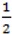 prior may be considered noninformative in the sense that it gives the same weight to all possible models it has been shown that this prior can be considered as informative since it puts more weight on models of size close to k/2 supporting a priori overparameterised and complicated models. This is especially problematic when k is large [37,38]. When meaningful prior information about *ψ* is unavailable, as is usually the case, perhaps the most reasonable strategy would be a fully Bayes approach that puts weak hyperior distributions on *ψ*. The potential drawback of this procedure is the computational limitation of visiting only a very small portion of the posterior when k is large yielding unreliable estimates of *ψ*. We defined the inclusion indicators as *ψ_g_* ~ *Bernoulli*(0.5) for three reasons: First, our set of covariates was small (k = 5) and it was very unlikely that this choice of prior affects BMA. Second, to minimise any possible tendency towards overparameterised models we implemented a two stage modelling strategy and eliminated covariates with small inclusion probability at the first stage. Third, MCMC computations for fully Bayesian models potentially impose high computational costs. By choosing conventional empirical Bayesian method we aimed to retain useful features of Bayesian variable selection in a pragmatic way.

In this paper we have compared Poisson, generalised Poisson and NB distributions for modelling count data when overdispersion is a problem. Results indicate that the Poisson distribution is not adequate to model cancer SIRs in our data setting. The negative binomial and the generalized Poisson distributions are more appropriate than the Poisson distribution. The negative binomial distribution and the generalized Poisson distributions are quite similar for the range of parameters in our study. It must be emphasized that for count data with small counts, various discrete distributions can fit the data sufficiently well [39].

When competing models exist, the information criterion such as Akaike information criterion (AIC), Bayes information criterion (BIC) and deviance information criterion (DIC) may be useful to select a single “best” model for final inference. However, these standard regression techniques and selection methods do not address the uncertainty associated with model specification. In contrast BMA considers a set of models with all available covariates. Then, it deals with the uncertainty in model form in the estimated parameters, which enables one to average across all models considering the posterior probabilities. Moreover, using the Gibbs sampler to search the model space for all possible models is efficient, due to limited number of covariates. We considered BMA in order to control the model uncertainty with respect to covariates. The advantages of using the BMA approach to account for model uncertainty have been assessed for several different classes of models [40,41,42]. Results from those studies showed that BMA improves predictive performance, by factors ranging from modest to substantial. Regarding the model uncertainty, we have considered only one component: which independent variables to include in the model. There are other components also such as uncertainty about functional forms of the independent variables, which can also be addressed by application of Bayesian methods but there is no evidence from prior work that this has led to improved predictive performance [43,44,45], and as such it was not attempted here.

## 5. Conclusions

The objectives of this study were to evaluate and compare the generalised Poisson and negative binomial models with the Poisson model commonly used for analysing count data. The results indicate that: (i) models with joint independence and spatial random effects were superior to the models with an independence random effect alone; (ii) models with alternative distributions that accommodate overdispersion performed better than Poisson regression. Using a spatial random effect term has the advantage of allocating the overdispersion to spatial and non-spatial components, recognizing the inherently spatial nature of the data. It was found in the case study that generalised Poisson or negative binomial models with conditional autoregressive correlation structure seemed to provide the most satisfactory basis for inference. The methodology presented is not specific to our example and can be applied in a variety of settings to produce more informative results than simple Poisson regression modelling.
